# Genetic characterization and antiretroviral resistance mutations among treatment-naive HIV-infected individuals in Jiaxing, China

**DOI:** 10.18632/oncotarget.15382

**Published:** 2017-02-16

**Authors:** Jinlei Guo, Yong Yan, Jiafeng Zhang, Jimei Ji, Zhijian Ge, Rui Ge, Xiaofei Zhang, Henghui Wang, Zhongwen Chen, Jianyong Luo

**Affiliations:** ^1^ Jiaxing Key Laboratory of Pathogenic Microbiology, Jiaxing Municipal Center for Disease Control and Prevention, Jiaxing 314001, PR China; ^2^ Institute of AIDS Control and Prevention, Zhejiang Provincial Center for Disease Control and Prevention, Hangzhou 310051, PR China

**Keywords:** HIV-1, treatment-naive, genotype, drug resistance, genetic diversity

## Abstract

The aim of this study was to characterize HIV-1 genotypes and antiretroviral resistance mutations among treatment-naive HIV-infected individuals in Jiaxing, China. The HIV-1 partial polymerase (pol) genes in 93 of the 99 plasma samples were successfully amplified and analyzed. Phylogenetic analysis revealed the existence of five HIV-1 genotypes, of which the most prevalent genotype was CRF01_AE (38.7%), followed by CRF07_BC (34.4%), CRF08_BC (16.1%), subtype B/B’ (5.4%), and CRF55_01B (2.1%). Besides, three types of unique recombination forms (URFs) were also observed, including C/F2/A1, CRF01_AE/B, and CRF08_BC/CRF07_BC. Among 93 amplicons, 46.2% had drug resistance-associated mutations, including 23.7% for protease inhibitors (PIs) mutations, 1.1% for nucleoside reverse transcriptase inhibitors (NRTIs) mutations, and 20.4% for non-nucleoside reverse transcriptase inhibitors (NNRTIs) mutations. Six (6.5%) out of 93 treatment-naive subjects were identified to be resistant to one or more NNRTIs, while resistance to NRTIs or PIs was not observed. Our study showed the genetic diversity of HIV-1 strains circulating in Jiaxing and a relative high proportion of antiretroviral resistance mutations among treatment-naive patients, indicating a serious challenge for HIV prevention and treatment program.

## INTRODUCTION

HIV-1 remains a global public health problem of unprecedented dimensions. According to the Joint United Nations Programme on HIV/AIDS (UNAIDS), there were 36.7 million (34.0 million-39.8 million) people living with HIV in 2015 [1]. Phylogenetic analysis allows classification of HIV-1 strains into four groups: M, N, O and P. The group M, responsible for the global HIV pandemic, has been further divided into nine subtypes (A-D, F-H, J and K) and 79 circulating recombinant forms (CRFs) to date (http://www.hiv.lanl.gov/content/sequence/HIV/CRFs/CRFs.html). Besides, a proportion of unique recombinant forms (URFs) have also been demonstrated. Actually, new CRFs and URFs continue to be identified and HIV diversity continues to increase [2]. The global distribution of HIV-1 genotypes is extremely complex and dynamic, and specific distributions of genotypes vary among different continents [3]. Globally, the most predominant subtype is subtype C that has spread to different continents, followed by subtype A and B; Subtype B dominates in North America, Western and Central Europe, the Caribbean, Latin America, and Australia [4]. All groups, subtypes and many CRFs have been reported in Africa [5]; The Middle East is mainly affected by subtype B and various CRFs; In India and Ethiopia the epidemics are nearly caused by subtype C. The epidemic in Eastern Europe and Central Asia is dominated by subtype A and B, while in South and Southeast Asia being CRF01_AE. In East Asia the epidemic is dominated by CRF07_BC, CRF08_BC, CRF01_AE and B [4].

Since antiretroviral treatment (ART) has been made available to AIDS patients, the number of patients received the ART has increased rapidly, an estimated 17 million people were accessing life-saving antiretroviral medicines at the end of 2015 according to UNAIDS [1]. The ART has reduced the morbidity and mortality associated with HIV infection, however the great success of ART is now threatened by HIV drug resistance [6]. The new ART recommendations (“treat all”) and the scale-up of pre-exposure prophylaxis [7] using antiretroviral drugs are likely to induce the HIV strains to mutate more quickly under the drug selection pressure. HIV drug resistance has already been observed among treatment-naive patients in China [8–10]. These reports merit attention that the prevalence of antiretroviral drug resistance may compromise the effect of current therapeutic regimens potentially or directly and stress the urgent need to intensify the routine implementation of HIV drug resistance surveys.

Located in the Yangtze River Delta region and northeast of Zhejiang province of China, Jiaxing is a city with very well-developed manufacturing industries attracting large numbers of migrant-workers each year. Since the first case of Jiaxing was detected in 1998, the HIV-1 infection rate has been increasing annually in this city. The HIV epidemiological survey of Jiaxing in 2015 showed that 255 newly diagnosed HIV-1 infected individuals came from 21 provinces as well as municipals, of which the migrants accounted for about 60% of the total infected cases, indicating infections among migrants are a big factor in the HIV-1 epidemic in this city. However, little is known on molecular epidemiology of HIV-1 in Jiaxing, thus our group examined genetic characteristics and antiretroviral resistance mutations among treatment-naive HIV-1 infected patients living in the city.

## RESULTS

### Subjects included

Of the 99 treatment-naive subjects, 93 (94.0%) pol genes were successfully amplified and sequenced. 81.0% of these subjects were male. Median age was 35 years (range: 16-72). Eighty-three subjects (89.2%) acquired HIV infection through sexual contact. More than half of the participants (56.0%) had the experience of marriage. Nineteen subjects received a high school education degree or above. Median CD4 cells count at sampling was 282 cells/mm^3^ (range: 12-1266) (Table 1).

**Table 1:** Demographic characteristics of study subjects ^a^ (n=93)

		Total n(%)^b^
Gender	Male	75(81.0%)
	Female	18(19.0%)
Household registered	Local residence in Jiaxing	40(43.0%)
	others	53(57.0%)
Age (years)	<18	2(2.2%)
	18-39	53(57.0%)
	40-59	28(30.1%)
	≥60	10(10.7%)
Marriage Status	Single	34(36.6%)
	Married	28(30.1%)
	Divorced/widowed	24(25.8%)
	Unknown	7(7.5%)
Education	Middle school and below	67(72.1%)
	High school and above	19(20.4%)
	Unknown	7(7.5%)
Occupation	Workers and peasants	39(41.9%)
	Commercial sex work	13(14.0%)
	Domestic workers and unemployed	25(27.0%)
	Employees in the service industry	4(4.3%)
	Students	2(2.1%)
	Others and unknown	10(10.7%)
Route of infection	Heterosexual transmission	50(53.7%)
	Homosexual transmission	33(35.5%)
	Intravenous drug use	4(4.3%)
	Unknown	6(6.5%)
CD4 cell count(cells/mm^3^)	≤200	27(29.0%)
	>200	66(71.0%)

a, those pol genes were successfully amplified and sequenced; b, values in brackets show the percentages of corresponding cases

### HIV-1 pol phylogenetic diversity

**Figure 1:**
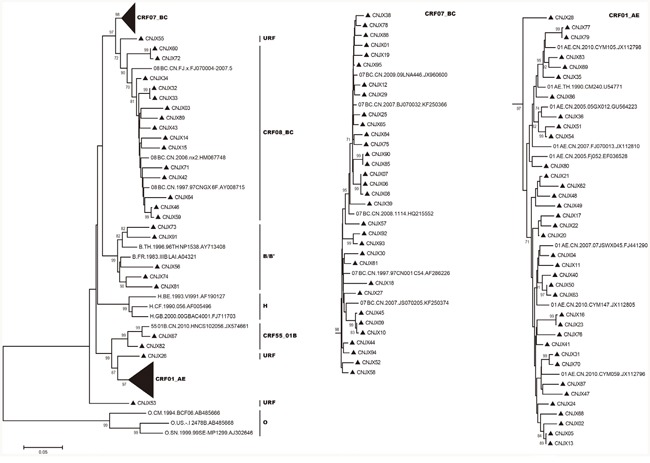
Phylogenetic trees of HIV-1 pol genes were constructed using MEGA 5 based on neighbor-joining methods The sample sequences and reference HIV-1 genotypes (subtypes B, B’, H, CRF01_AE, CRF07_BC, CRF08_BC, CRF55_01B and group O) were aligned using Bioedit with minor manual adjustments. The statistical robustness of the neighbor-joining tree and reliability of the branching patterns were confirmed by one thousand bootstrap replicates. Only bootstraps greater than 70% are shown at each node. All samples analyzed in the trees are shown by black triangles (▴). URF represents unique recombinant forms. The scale bar represents 5% nucleotide sequence divergence.

**Figure 2:**
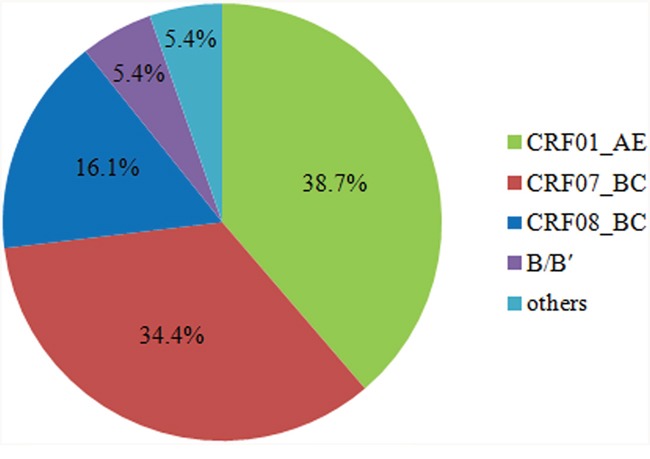
Genotype distribution of HIV-1 strains among treatment-naive individuals in Jiaxing The partial pol gene fragments of 93 HIV-1 patients were amplified and sequenced. These genotypes were determined by the phylogenetic analysis with reference HIV-1 genotypes. The distribution and percentages of HIV-1 genotypes were shown in pie chart.

**Figure 3:**
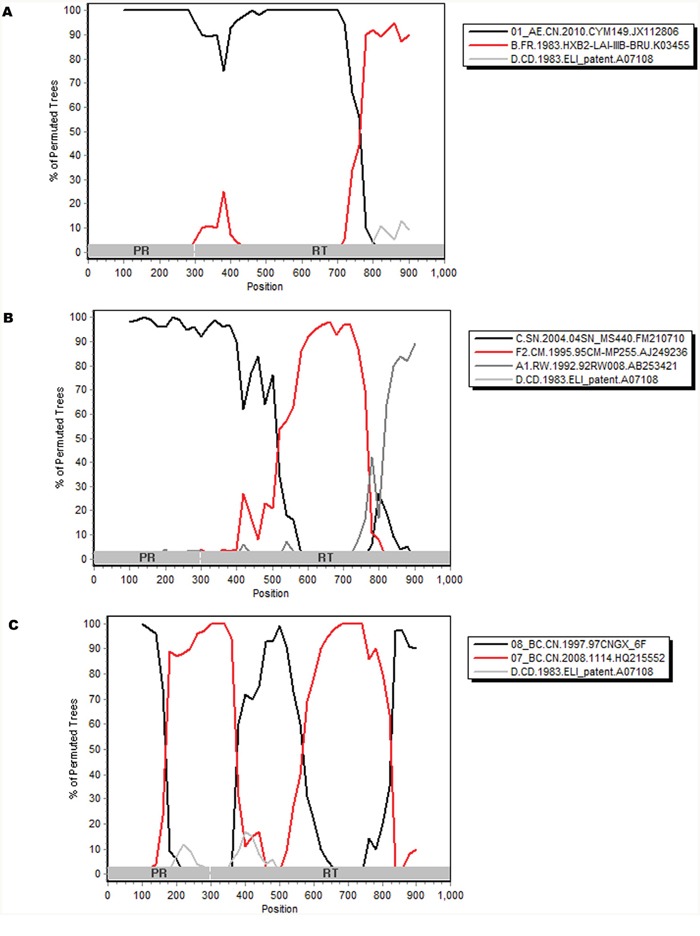
Bootscanning analyses of the partial pol genes of three new unique recombinant strains CNJX26 A., CNJX53 B., CNJX55 C A bootscanning plot was constructed by using Simplot 3.5.1, which was based on 100 replicates with a 200-bp sliding window moving in steps of 20 bases. The horizontal axis is the nucleotide position from the 5’ end of the analyzed sequence, the vertical axis indicates the percentage supporting the clustering with reference sequences of the analyzed sequence. PR, protease; RT, reverse transcriptase; Subtype references were A1, B, C, D, F2, CRF01_AE, CRF07_BC, CRF08_BC which were shown at the upper right of the figure.

Phylogenetic trees based on pol genes revealed that a majority of the HIV-1 isolates (36, 38.7%) studied belonged to the CRF01_AE, followed by CRF07_BC (32, 34.4%), CRF08_BC (15, 16.1%), subtype B/B’ (5, 5.4%), CRF55_01B (2, 2.1%) (Figure 1, Figure 2). The jpHMM-HIV and bootscanning analyses were performed on three sequences, which did not cluster with any present known references. The results revealed the presence of C/F2/A1 intersubtype recombinant strains with different mosaic structures from those of A1, C and F2 in one sample (CNJX53), the second recombination (CNJX26) was among the CRF and subtype, i.e. CRF01_AE/B, while the third (CNJX55) was among CRFs, i.e. CRF08_BC/CRF07_BC (Figure 3). The sequence analysis revealed a broad diversity in the samples studied.

The distribution of HIV-1 strains was uneven among different risk groups. CRF08_BC strains were more likely to be found among heterosexually infected individuals (12, 80%); While most CRF01_AE and CRF07_BC strains were detected in two groups: homosexually infected individuals (20, 55.6%), (10, 31.3%) and heterosexually infected individuals (14, 38.9%), (18, 56.3%) respectively (Table 2).

**Table 2:** Distribution of HIV-1 genotypes in different risk groups

Risk group	B/B’ (n=5)	CRF01_AE (n=36)	CRF07_BC (n=32)	CRF08_BC (n=15)	CRF55_01B (n=2)	URF ^b^ (n=3)
Heterosexual contact	4 (80%)	14 (38.9%)	18 (56.3%)	12 (80%)	1 (50%)	1 (33.3%)
Homosexual contact	1 (20%)	20 (55.6%)	10 (31.3%)	0 (0%)	1 (50%)	1 (33.3%)
IDU ^a^	0 (0%)	0 (0%)	2 (6.3%)	1 (6.7%)	0 (0%)	1 (33.3%)
Unknown	0 (0%)	2 (5.5%)	2 (6.3%)	2 (13.3%)	0 (0%)	0 (0%)

a, Intravenous Drug Use; b, Unique Recombinant Form

### Genotypic analysis of HIV-1 drug resistance

Pol genes were successfully amplified from 93 HIV positive samples and were subjected to drug resistance analysis including PI, NRTI and NNRTI resistance-associated mutations through the Stanford HIV Drug Resistance Database. Among our subjects, 46.2% of the amplicons (43/93) contained drug resistance-associated mutations, including 23.7% (22/93) for PI mutations, 1.1% (1/93) for NRTI mutations, and 20.4% (19/93) for NNRTI mutations. PI mutations, such as T74S, L10I, A71T/V, K20I, V11I, were all minor mutations which by themselves showed no significant effect on phenotypes, but might affect the progress of drug resistance or improve replication of viruses containing major mutations [14]. Among samples with reverse transcriptase inhibitors (RTIs) resistance mutations, only one sample harbored NRTI resistance mutation, i.e. T69N, which did not give any resistance to NRTIs; Among samples with NNRTI resistance mutations, the most frequent amino acid substitutions were at the 138^th^ and 179^th^ codon of the reverse transcriptase gene, such as the E138A/K/G (3.2%, 3/93), V179D/E/A/T mutation (9.7%, 9/93), all were rilpivirine (RPV) resistance-associated major or minor mutations [15] (Table 3); Overall, only six individuals (6.5%) showed low to high-level resistance to efavirenz (EFV), etravirine (ETR), rilpivirine (RPV), nevirapine (NVP), all were NNRTIs (Table 4); K103N, conferring a high-level resistance to NVP and EFV, found in three (50%, 3/6) samples, was one of the most common NNRTI-related mutations;

**Table 3:** HIV-1 antiretroviral resistance mutations identified among different genotypes in treatment-naive subjects

Genotype	PI (%) ^a^	PI mutation	NRTI (%) ^a^	NRTI mutation	NNRTI (%) ^a^	NNRTI mutation
CRF01_AE	19.4%(7/36)	V11I(1),T74S(1), L10I/V(3), K20I(2)	2.8%(1/36)	T69N(1)	22.2%(8/36)	V90I(2), V106I(1), V179D(5), K103N(1)
CRF07_BC	31.3%(10/32)	L10I(5), A71V/T(5)	0%(0/32)	-	9.4%(3/32)	V179D(1), K103N(2)
CRF08_BC	6.7%(1/15)	L10I(1)	0%(0/15)	-	33.3%(5/15)	V179D(1), E138A/K(3), V90I(1)
B/B’	60%(3/5)	L10I(1), A71T(3)	0%(0/5)	-	20%(1/5)	V106I(1)
CRF55_01B	0%(0/2)	-	0%(0/2)	-	100%(2/2)	V179E(2)
URF	33.3%(1/3)	A71T(1)	0%(0/3)	-	0%(0/3)	-
Total	23.7%(22/93)	-	1.1%(1/93)	-	20.4%(19/93)	-

PI, protease inhibitor; NRTI, nucleoside reverse transcriptase inhibitor; NNRTI, non-nucleoside reverse transcriptase inhibitor; a, the percentage of the samples harboring the PI or NRTI or NNRTI-related mutations in the specific genotype; - : denotes the absence of mutations.

**Table 4:** Characteristics of patients with primary resistance to antiretroviral drugs

Sample ID	Sex/age(years)/subtype	Route of infection/diagnosis year	Resistance mutation			Resistance profile		
			**PI**	**NRTI**	**NNRTI**	**Low**	**Intermediate**	**High**
CNJX14	M/42/CRF08_BC	Hetero/2015	-	-	E138K,V179AT	EFV, ETR, NVP	RPV	
CNJX15	F/39/CRF08_BC	Hetero/2015	-	-	E138A	RPV		
CNJX51	F/68/CRF01_AE	Hetero/2015	-	-	K103N			EFV,NVP
CNJX61	M/34/CRF07_BC	IDU/2012	-	-	K103N			EFV,NVP
CNJX84	M/39/CRF07_BC	MSM/2015	-	-	K103N			EFV,NVP
CNJX89	M/56/CRF08_BC	IDU/2016	-	-	E138A	RPV		

M, male; F, female; Hetero, Heterosexual contact; MSM, men who have sex with men; IDU, intravenous drug use; EFV, Efavirenz; ETR, Etravirine; RPV, Rilpivirine; NVP, Nevirapine; - : denotes the absence of mutations against the corresponding drugs

## DISCUSSION

Our study firstly detected HIV-1 subtypes and CRFs currently circulating in Jiaxing among treatment-naive individuals. HIV-1 subtype B/B’, CRF01_AE, CRF07_BC, CRF08_BC and CRF55_01B were identified, of which CRF01_AE was the predominant genotype, consistent with the previous study in Zhejiang province [16]. Three URFs, i.e. CRF01_AE/B, C/F2/A1, CRF08_BC/CRF07_BC were also identified, of which two subjects were migrant workers, one was an intravenous drug user from Yunnan province (data not shown). The number of possible URFs detected may be underestimated in this study because only the partial pol gene is sequenced providing limited information on recombination. The identification of CRFs and URFs indicated the high genetic diversity of HIV-1 strains circulating in Jiaxing. Genetic variability of HIV results from the high mutation and recombination rates, together with high replication capacity of the virus [17, 18]. Of note is that recombination requires coinfection or superinfection of viral strains within an individual [19], indicating that coinfection and/or superinfection occur at an alarming speed in Jiaxing. Besides, the migration of HIV-1 infected individuals can also promote viral recombination. Therefore, for Jiaxing, a city with lots of migrant workers flowing into, it is important to identify the genotype to monitor the dynamics and complexity of the HIV epidemic.

Since 1980s, about 40 kinds of antiretroviral drugs have been approved by US Food and Drug Administration, which greatly decreased the morbidity and mortality of HIV infection [20]. However, HIV drug resistance in treatment-naive individuals is increasing worldwide, which may result from the transmission of emergent drug-resistant virus strains to treatment-naive individuals [21]. Our observed drug resistance prevalence (6.5%) in treatment-naive individuals is classified as a moderate level (5%-15%) according to the WHO classification [22], which was similar to the drug resistance prevalence in the recent HIV infections in Zhejiang province, China [8], higher than transmitted drug resistance rates between 2004 (2.9%) and 2005(4.4%) in a nationwide investigation of China when the main transmission route was blood donation [23], lower than those in US and Europe where rates were reported to be 14.6% (2006) [24] and 8.9% (2002-2007) [25], South African with pre-treatment HIV-1 resistance prevalence of 9.0% during 2013 to 2014 [26].

Currently, the most common regimen for HIV patients in Jiaxing contains a combination of a NNRTI plus two NRTIs, such as tenofovir disoproxil fumarate (TDF) / azidothymidine (AZT) + lamivudine (3TC) +EFV/NVP. It was noteworthy that in our study all six patients were resistant to NNRTIs (EFV, ETR, RPV, NVP), while resistance to NRTIs or PIs was not observed. Previous study showed that NNRTIs had a low genetic barrier to drug resistance, a single amino acid change might be sufficient for high-level drug resistance, such as K103N, which had limited effect on viral replication and persisted long after transmission [27, 28]. Besides, NNRTIs usually have longer plasma half-lives than NRTIs [29], making it remain longer in plasma than NRTIs once a patient stops taking drugs, thus induce drug resistance mutations more easily; The above reasons may help explain why all resistant subjects were against NNRTIs. Besides, researches showed that NNRTIs-related mutations increased significantly among newly diagnosed HIV-infected patients in Europe [25, 30], implying us to pay attention to NNRTIs-related mutations. Surveillance of drug resistance mutations among treatment-naive patients will be necessary, thus European and USA guideline panels have already recommended drug-resistance testing prior to treatment [31, 32]. It is hoped that in the future baseline genotypic drug resistance testing can be performed in China to guide doctors select the optimal regimen for a particular patient.

One limitation of our study is that we used standard dideoxynucleotide sequencing, although used as a standard method in HIV drug-resistance testing, fails to identify drug-resistance minority variants that are below 20% of the virus population [33], those minorities can be clinically important as they may grow under drug pressure and lead to treatment failure, thus the method probably underestimates the real prevalence of resistance mutations among treatment-naive patients.

The high genetic diversity of HIV-1 strains and a relative high proportion of antiretroviral resistance mutations among treatment-naive patients pose a serious challenge for HIV prevention and treatment program in Jiaxing; It was noteworthy that most subjects had relatively low levels of education, therefore strengthening propaganda and education of HIV prevention knowledge would be very necessary. This is the first cross-sectional survey describing the genetic characterization of HIV-1 and patterns of PI, NRTI and NNRTI resistance-associated mutations among treatment-naive patients living in Jiaxing, which may provide some implications for future HIV prevention and antiretroviral treatment strategies.

## MATERIALS AND METHODS

### Study subjects

A total of 104 HIV-1 infected patients living in Jiaxing were enrolled continuously from April 2015 to February 2016. HIV-1 infection in these patients was confirmed by western blot. Of which 5 patients received ART, the remaining 99 subjects were self-reported to be treatment-naive. The epidemiology data were collected by trained interviewers. The study was approved by the Review Board of Jiaxing Municipal Center for Disease Control and Prevention and conducted according to the principles of the World Medical Association Declaration of Helsinki. All subjects provided written informed consent prior to participating in this study. The participants were simultaneously informed of their right to repeal consent by them or their kin, guardians, or caretakers.

### HIV-1 pol gene amplification and phylogenetic analysis

Subjects’ whole blood samples were collected and CD4 cell count was measured within 24 hours after sampling. Meanwhile, plasma was separated by centrifugation and stored at −80°C until use. HIV-1 polymerase (pol) gene (containing the full length protease (PR) gene and the first 300 codons of the reverse transcriptase (RT) gene) was amplified, purified and sequenced as previously described [11]. Each sequence was submitted to the Los Alamos National Laboratory HIV Sequence Database (http://www.hiv.lanl.gov/content/index) and COMET HIV-1 [12] to determine HIV genotype, further confirmed by phylogenetic analysis using standard reference sequences. To identify the possible recombination, the sequences were submitted to Jumping Profile Hidden Markov Model (jpHMM-HIV) software [13], and confirmed by bootscanning analyses using Simplot 3.5.1.

### Genotypic baseline of HIV-1 drug resistance

For analysis of HIV-1 drug resistance mutation, each sample sequence was submitted to the Stanford HIV Drug Resistance Database (http://hivdb.stanford.edu) to detect drug-resistance mutations including protease inhibitor (PI) major and minor resistance mutations, nucleoside reverse transcriptase inhibitor (NRTI) and non-nucleoside reverse transcriptase inhibitor (NNRTI) resistance mutations. Genotypic susceptibility was interpreted by HIVdb program.
